# Actinomycetoma in SE Asia: the first case from Laos and a review of the literature

**DOI:** 10.1186/1471-2334-12-349

**Published:** 2012-12-12

**Authors:** Sayaphet Rattanavong, Sivay Vongthongchit, Khamhou Bounphamala, Phouvong Vongphakdy, Jacques Gubler, Mayfong Mayxay, Rattanaphone Phetsouvanh, Ivo Elliott, Julie Logan, Robert Hill, Paul N Newton, David Dance

**Affiliations:** 1 Lao-Oxford-Mahosot Hospital-Wellcome Trust Research Unit, Microbiology Laboratory, Mahosot Hospital, Vientiane, Lao PDR; 2 Surgery Unit, Xiengkhuang Provincial Hospital, Xiengkhuang, Lao PDR; 3 General Surgery Department, Mahosot Hospital, Vientiane, Lao PDR; 4 Department of Medicine, Infectious Diseases, Kantonsspital, Winterthur, Switzerland; 5 Faculty of Postgraduate Studies, University of Health Sciences, Vientiane, Lao PDR; 6 Centre for Clinical Vaccinology and Tropical Medicine, Nuffield Department of Clinical Medicine, University of Oxford, Churchill Hospital, Oxford, OX3 7LJ, UK; 7 Department for Bioanalysis and Horizon Technologies, Health Protection Agency Microbiology Services Colindale, 61 Colindale Avenue, London, NW9 5HT, UK; 8 Antibiotic Resistance Monitoring & Reference Laboratory (ARMRL), Health Protection Agency Microbiology Services Colindale, 61 Colindale Avenue, London, NW9 5EQ, UK

**Keywords:** Mycetoma, Madura foot, *Actinomadura madurae*, Lao PDR, Laos, Southeast Asia

## Abstract

**Background:**

Mycetoma is a chronic, localized, slowly progressing infection of the cutaneous and subcutaneous tissues caused either by fungi (eumycetoma or implantation mycosis) or by aerobic actinomycetes (actinomycetoma). It is acquired by traumatic implantation, most commonly in the tropics and subtropics, especially in rural agricultural communities. Although well recognized elsewhere in Asia, it has not been reported from the Lao People’s Democratic Republic (Laos).

**Case presentation:**

A 30 year-old female elementary school teacher and rice farmer from northeast Laos was admitted to Mahosot Hospital, Vientiane, with a massive growth on her left foot, without a history of trauma. The swelling had progressed slowly but painlessly over 5 years and multiple draining sinuses had developed. Ten days before admission the foot had increased considerably in size and became very painful, with multiple sinuses and discharge, preventing her from walking. Gram stain and bacterial culture of tissue biopsies revealed a branching filamentous Gram-positive bacterium that was subsequently identified as *Actinomadura madurae* by 16S rRNA gene amplification and sequencing*.* She was treated with long-term co-trimoxazole and multiple 3-week cycles of amikacin with a good therapeutic response.

**Conclusion:**

We report the first patient with actinomycetoma from Laos. The disease should be considered in the differential diagnosis of chronic skin and bone infections in patients from rural SE Asia.

## Background

Mycetoma, commonly known as Madura foot, is a chronic, localized, slowly progressive, granulomatous infection of the deep dermis and subcutaneous tissue caused either by fungi (eumycetoma or implantation mycosis) or by aerobic actinomycetes (actinomycetoma) [1]. It was first described in India by Gill in 1842 and the term mycetoma was first used by Carter in 1860 [1,2]. Mycetoma occurs unevenly worldwide but is endemic in tropical and sub-tropical regions, particularly between latitudes 15° S and 30° N, known as the ‘Mycetoma belt’. The belt includes Sudan, Somalia, Senegal, India, Yemen, Mexico, Venezuela, Colombia and Argentina. In Sudan, 1231 cases were reported within a 2.5-year period and it was found to be the third commonest cause of amputation [3,4]. Cases imported from endemic areas are occasionally seen in temperate countries [5].

More than 20 species of bacteria and fungi have been identified as aetiologic agents of mycetoma: approximately 60% of cases are due to bacteria and 40% are caused by fungi. The predominant cause varies geographically, probably because of environmental factors [6]. In India, Mexico and Brazil, actinomycetoma is the most common but in Sudan most cases are implantation mycoses. The agents most often responsible for causing actinomycetoma are *Actinomadura madurae*, *Actinomadura pelletieri*, *Nocardia brasiliensis* and *Streptomyces somaliensis*. *Madurella mycetomatis*, *M. grisea* and *Pseudallescheria boydii* are the most common causes of implantation mycoses [7-11]. Actinomycetoma tends to progress more rapidly, with greater inflammation and tissue destruction and earlier invasion of bone than implantation mycosis. Initially, the patient may feel pain or discomfort at the inoculated site. After traumatic inoculation, painless subcutaneous nodules slowly develop and spread. Later, the nodules increase in size and quantity with accompanying sinuses that drain serous, sero-sanguineous or purulent fluid [12]. The main route of infection is uncertain but, as organisms are usually present in the soil [13], it is likely that they are implanted into the host tissue by traumatic inoculation such as thorn pricks or splinters, although only half of the patients can remember a history of trauma preceding the disease [7]. This theory is supported by the facts that cases are common among farmers, herdsmen, field labourers or people walking bare foot and that the main affected areas are foot and hand, although other parts may be involved less frequently [12,14-16]. The incubation period is variable, from three months to nine years in natural infections. Since the mean duration before the first medical evaluation is five years, patient recall of trauma may often be unreliable [7,17].

Mycetoma is more common in males than females, with an overall sex ratio of 3:1, possibly relating to differences in occupational and other outdoor activities rather than gender differences in susceptibility [11,15]. The condition is also commonest in young adults (16–40 years old) [9].

Irrespective of the causative agent, the clinical presentation is similar, with tissue swelling and draining sinuses that usually discharge visible grains. It is important to determine the causative agent of mycetoma in order to choose the appropriate treatment. The colour of the grains in the discharge from the sinuses may be helpful for presumptive identification of the aetiology, although this is not entirely reliable. Black grains are said to be associated with *Madurella mycetomatis*, *Madurella grisea*, *Exophiala jeanselmei*, *Curvularia* species, *Leptosphaeria* species and *Pyrenochaeta* species, white grains with *Nocardia* species, *Actinomadura madurae*, *Pseudallescheria boydii*, *Acremonium* species, *Cylindrocarpon* species and *Fusarium* species, yellow grains with *Streptomyces somaliensis* and red with *Actinomadura pelletieri*[3,14]. Differential diagnosis includes soft tissue tumours such as lipoma, fibroma, sarcoma, malignant melanoma and chronic osteomyelitis caused by pyogenic bacteria or mycobacteria, and other mycoses such as chromoblastomycosis, lobomycosis, paracoccidioidomycosis, phaeohyphomycosis and sporotrichosis [18].

Although cases have been reported from neighbouring countries, mycetoma has never been reported in Laos: we report here the first case.

## Case presentation

**Figure 1:**
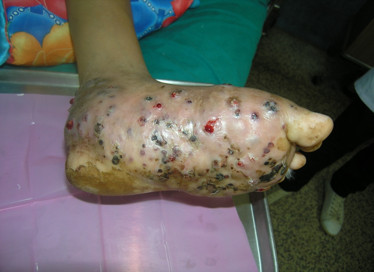
Left foot of the patient showing classic features of mycetoma: tumefaction, sinus tracts and discharge.

**Figure 2:**
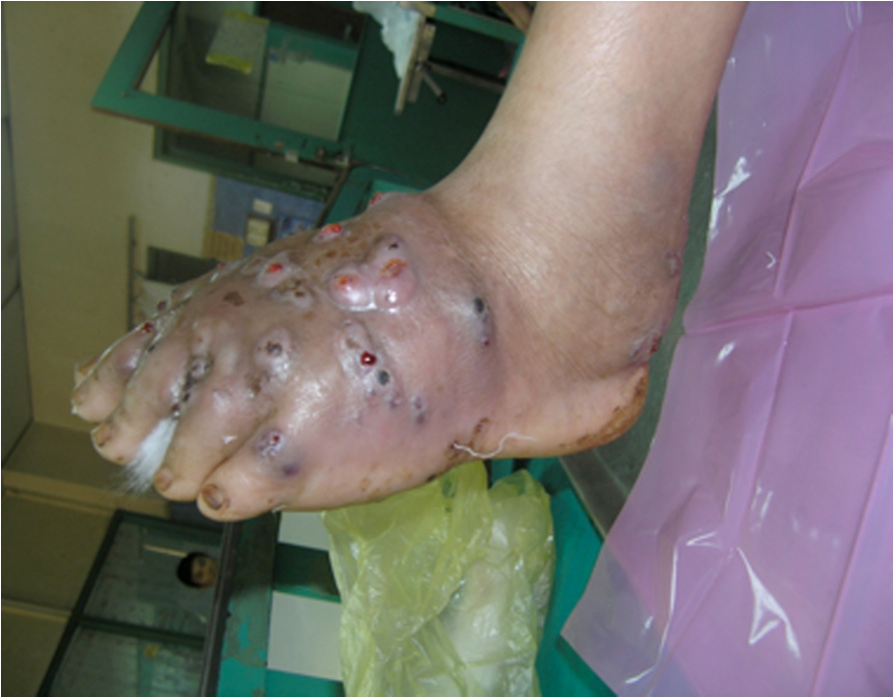
Left foot of the patient showing classic features of mycetoma: tumefaction, sinus tracts and discharge.

**Figure 3:**
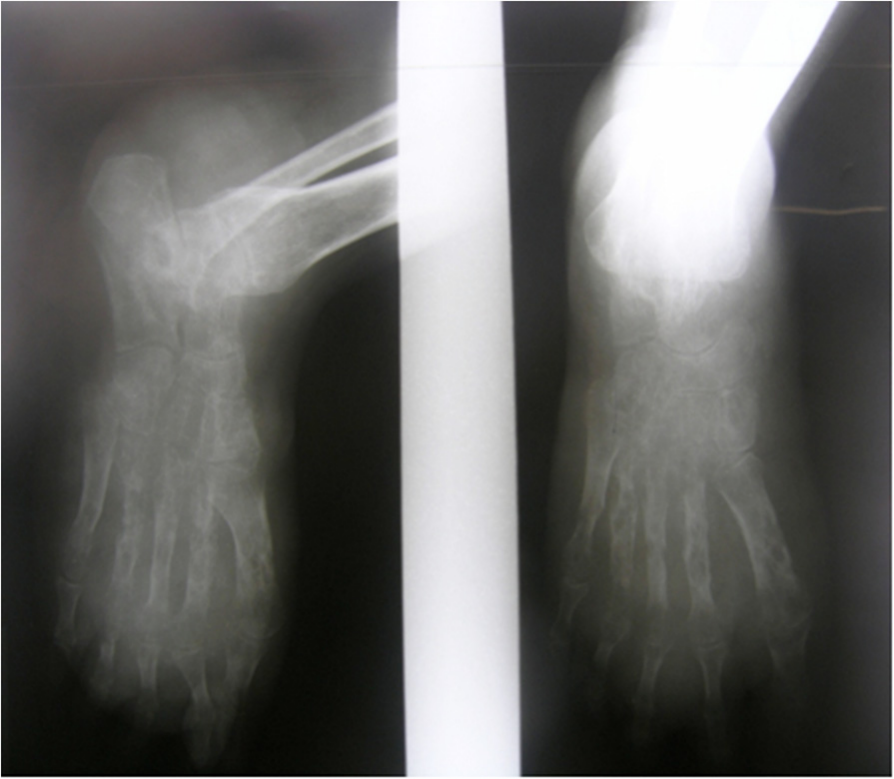
X-ray of the left foot showing sign of destruction of the metatarsal bones.

**Figure 4:**
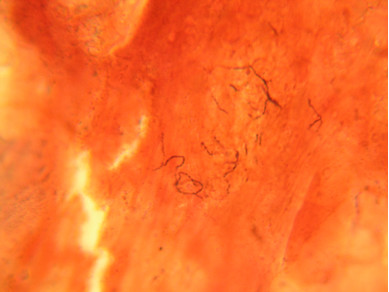
Gram stain of smear from left foot tissue biopsy revealed branching, filamentous Gram positive bacteria.

**Figure 5:**
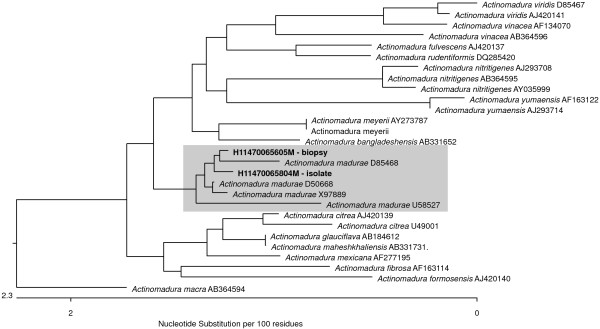
**Unrooted tree of *Actinomadura *spp. partial 16S rRNA sequences (bases ~1200 bp, accession numbers after species name).**

**Figure 6:**
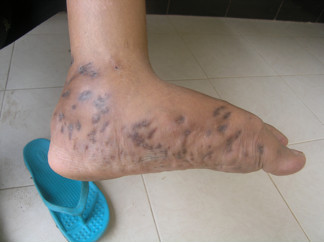
Left foot of the patient after 5 cycles of amikacin showing considerable resolution of swelling and healing of sinus tracts.

**Figure 7:**
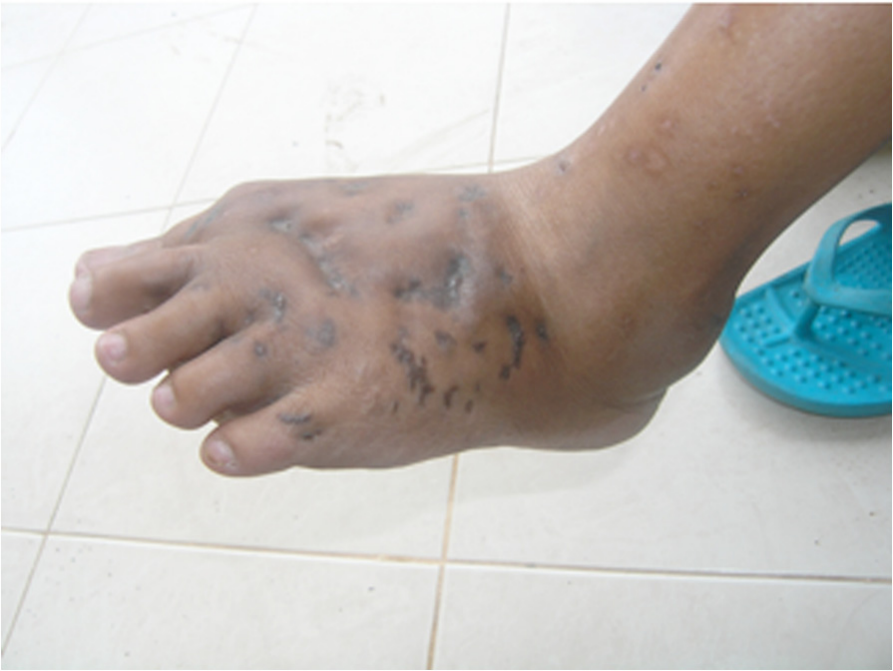
Left foot of the patient after 5 cycles of amikacin showing considerable resolution of swelling and healing of sinus tracts.

**Figure 8:**
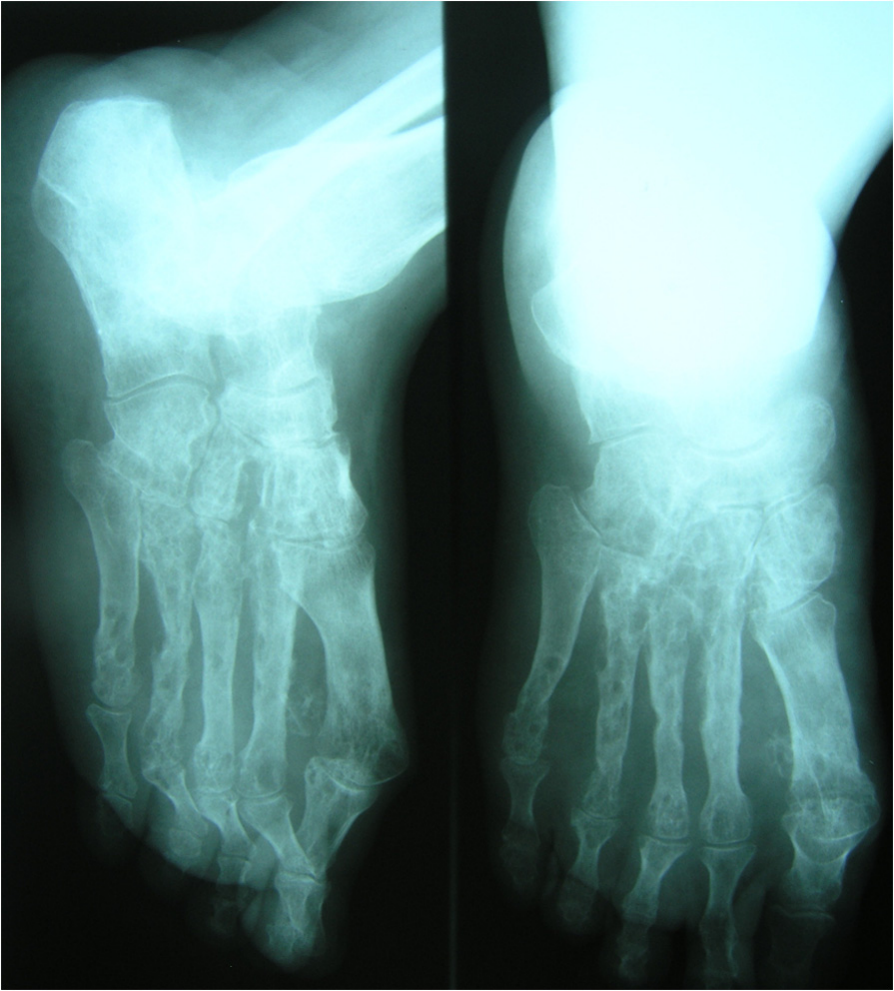
X-ray of the left foot after 1 year of treatment showing considerable residual bony abnormalities.

An otherwise healthy 30-year-old female elementary school teacher and rice farmer from Namun village (19.6552435^·^° N, 103.5781784^·^° E), Kham District, Xiengkhuang Province, northeast Laos, presented to Mahosot Hospital in October 2011 with tumefaction, pain and multiple draining sinuses on her left foot. The lesion had started 5 years previously, initially with an uncomfortable feeling and itching on the left plantar surface that over a few days became indurated and developed a spontaneously draining sinus. She remembered no specific injury to her foot. Over the next five years, the lesion slowly and painlessly progressed, with swelling and multiple draining sinuses. Ten days before admission she developed acute swelling of the foot with severe pain associated with multiple sinuses and discharge from the foot that prevented her from walking. Foot X-ray done at a local hospital showed signs of osteolysis of the left metatarsal bones. With a diagnosis of chronic osteomyelitis of the left foot she was treated with oral cloxacillin for a few days, but when branching Gram positive rods were demonstrated on a Gram stain of an aspirate from the lesion at the provincial hospital she was transferred to Mahosot Hospital. On examination, she was orientated, afebrile with normal vital signs, and physical examination was unremarkable except for a massive tumour-like lesion of the left foot with multiple sinuses on the dorsal and plantar surfaces. The overlying skin was moderately erythematous and was tender and painful on palpation with evidence of a sero-sanguinous discharge containing no obvious grains (Figures 1 and 2). There was no regional lymphadenopathy or lymphangitis present. Admission laboratory investigation (normal ranges in parentheses) revealed a total white blood cell count of 6.1 × 10^9^/L (6.0-8.0 × 10^9^/L), 68% neutrophils (45-70%), haematocrit 28% (37-47%), haemoglobin 75 g/L (120–160 g/L), mean corpuscular volume 59 fL (80-95fL), platelets 449 × 10^9^/L (150-300 × 10^9^/L), creatinine 72 μmol/L (53-120 μmol/L), urea 4.3 mmol/L (5.3-16 mmol/L), aspartate transaminase 50 U/L (0–37 U/L), alanine transaminase 34 U/L (0–45 U/L) and blood glucose 4.2 mmol/L (4.1- 6.4 mmol/L). A radiograph of the left foot confirmed partial osteolysis of the metatarsal bones (Figure 3). Four 3 mm diameter tissue biopsies were taken from her left foot under local anaesthesia. Smears from these samples underwent microscopy after Gram stain, Ziehl-Neelsen stain and the 20% potassium hydroxide technique. The samples were cultured aerobically on blood, chocolate, MacConkey, Sabouraud and Ashdown’s agars and were submitted for mycobacterial culture and histopathology. The Gram stain revealed scanty branching filamentous Gram positive bacteria (Figure 4), which grew after 5 days’ incubation on chocolate and blood agar. She was presumptively diagnosed as suffering from actinomycetoma and started on amikacin (15 mg/kg/day) combined with trimethoprim-sulfamethoxazole (7/35 mg/kg/day), both in two daily doses. One biopsy sample, which had been immediately frozen at −80°C, and the organism were both identified by 16S rRNA amplification and sequencing as *Actinomadura madurae* (Figure 5) by the Health Protection Agency (HPA), UK [19]. Broth dilution MICs for the organism, performed by the HPA, gave the following results (mg/l): amikacin ≤ 0.25 (S), gentamicin 2 (S), tobramycin ≤ 0.25 (S), amoxicillin/clavulanate 4 (S), cefotaxime 4, ceftriaxone 0.25 (S), imipenem ≤ 0.125 (S), meropenem 0.5 (S), co-trimoxazole 0.032 (S), clarithromycin 4 (I), ciprofloxacin ≤ 0.25 (S), moxifloxacin ≤ 0.25 (S) and doxycycline ≤ 0.25 (S). After 21 days of intravenous amikacin and oral trimethoprim-sulfamethoxazole, the patient’s left foot showed some evidence of healing with decreasing pain, swelling and discharge without any side effects from treatment. She was transferred for further treatment at a district hospital close to her home. On the basis of published recommendations, amikacin was given for sequential cycles of 3 weeks with 2 week breaks, and trimethoprim-sulfamethoxazole was continued throughout: creatinine and hearing were monitored weekly without abnormalities [20,21]. After 5 cycles of treatment there had been a marked reduction in swelling (Figures 6 and 7), considerable symptomatic improvement, and the patient was able to walk and to return to teaching. The patient continued on oral co-trimoxazole alone until a total of 12 months of treatment had been completed, at which stage she had no pain at rest and there were no sinuses or discharge from the foot, although there was still considerable residual bony abnormality evident radiologically (Figure 8).

Our patient represents a fairly typical, albeit severe, case of actinomycetoma caused by *A. madurae*, with involvement of the underlying bone. Bone involvement is common in advanced stages of the disease (an incidence of 73% was reported in one series), leading to deformity and disability of the affected area [22].

Even a classic clinical presentation and typical colours of grains in mycetoma does not reliably distinguish actinomycetoma from implantation mycosis, so laboratory investigations, including microscopy, histology and extended cultures are indicated. Other investigations including fine needle aspiration cytology, imaging (X-ray, magnetic resonance and ultrasound), sero-diagnosis (counter-immunoelectrophoresis and ELISA) and molecular detection and identification may also be useful [14,18]. Although the evidence base is poor, without clinical trials, combined treatment with multiple antibiotics is usually recommended in order to prevent drug resistance and eradicate any residual infection. Reported cure rates vary widely ranging from 60% to 90%. Sulphonamides and sulphonamide combinations are recommended as the first line treatment. Aminoglycosides, tetracyclines, rifampicin, ciprofloxacin and amoxicillin-clavulanate have also been used successfully. The combination of amikacin and co-trimoxazole, the former given in cycles, has produced excellent clinical results, especially in those at risk of pulmonary spread or vertebral involvement [21]. Five to ten cycles may be needed to achieve cure. Furthermore, surgery may be considered for small lesions, infections that fail to respond to antibiotics and to help medical treatment by reduction of the infective load or deal with life-threatening lesions [12,14,18,20].

This is the first case of mycetoma reported from Laos. Cases have been reported infrequently from Southeast Asia, the majority of which has a prolonged rainy season unlike the arid regions usually associated with mycetoma. Mycetoma has, however, been reported from Singapore, Malaysia, Philippines, Indonesia, Cambodia, Thailand and Vietnam. Table 1 shows cases identified by searching PubMed using the terms ‘Mycetoma’ AND ‘Southeast Asia’ or ‘Mycetoma’ AND the names of individual countries (‘Lao’, ‘Thai’, ‘Cambodia’, ‘Burma’, ‘Myanmar’, ‘Malaysia’, ‘Singapore’, ‘Indo’, ‘Philip’, ‘Timor’, ‘Brunei’, ‘Vietnam’). The true incidence in the region, however, is unknown.

**Table 1:** Summary of reports of Mycetoma in Southeast Asia

**Country**	**Year**	**Patients**	**Diagnosis**	**Organism**^**1**^	**Reference**
Singapore	1967	1	Histology	*Monosporium apiospermum*	[23]
Malaysia	1968	1	Histology and culture	*Phialophora jeanselmei*	[24]
	1969	1	Histology	*Madurella mycetomi*	[25]
	1982	1	Histology and culture	*Streptomyces somaliensis*	[26]
Philippines	1960	1	Culture	*Madurella grisea*	[27]
	1965	1	Histology and culture	*Madurella grisea*	[28]
Indonesia	1938	1	Culture	*Madurella tropicana*	[29]
	1978	14	NA^2^	NA	[30]
Cambodia	1963	1	Histology and culture	*Pyrenochaeta romeroi*	[31]
	1965	1	Grains and radiography	*Madura mycetes*	[32]
Thailand	1981	17	Histology and culture	*Nocardia asteroides* (6 cases); *N. caviae* (2 cases); *N. brasiliensis* (2 cases); *N. rosatii* (1 case); *Madurella mycetomii* (3 cases); *A. boydii* (1 case); *P. jeanselmei* (1 case) and *Strep. madurae* (1 case).	[33]
	1994-1997	14	Histology and culture	Only one case was confirmed by culture as *Cladosporium carrionii*	[34]
Vietnam	1973	1	Culture	*Nocardia otitidiscaviarum*	[35]

NOTES

1. The nomenclature used in the original publications has been retained. In several cases this was a presumptive identification based on histology alone.

2. NA: not available.

## Conclusions

This patient confirms the existence of mycetoma caused by *Actinomadura madurae* in Laos. Approximately 78% of the Lao population works mainly in agriculture and there are, as elsewhere in the rural tropics, few microbiology laboratories, making it likely that the condition is under-diagnosed. Isolation of the causative organism enabled medical treatment, which almost certainly saved this young woman from amputation and lifelong disability. Mycetoma should be considered in the differential diagnosis of chronic skin and bone infections in Laos and elsewhere in Southeast Asia.

## Consent

Written informed consent was obtained from the patient for publication of this Case report and any accompanying images. A copy of the written consent is available for review from the Series Editor of this journal.

## Competing interests

All authors declare that they have no competing interest.

## Authors’ contributions

SR wrote the manuscript with DD, who made the laboratory diagnosis. JG, KB and SV made the initial clinical diagnosis and referred the patient for further management. SR, SV, IE, MM, RP, PN and DD helped with patient management. JL identified the organism by 16S rRNA amplification and sequencing and RH undertook the susceptibility testing. All authors were involved in revising the manuscript and approved the final version.

## Pre-publication history

The pre-publication history for this paper can be accessed here:

http://www.biomedcentral.com/1471-2334/12/349/prepub
